# Reasons for Nonuse, Discontinuation of Use, and Acceptance of Additional Functionalities of a COVID-19 Contact Tracing App: Cross-sectional Survey Study

**DOI:** 10.2196/22113

**Published:** 2022-01-14

**Authors:** Michel Walrave, Cato Waeterloos, Koen Ponnet

**Affiliations:** 1 MIOS Research Group and GOVTRUST Centre of Excellence Department of Communication Studies, Faculty of Social Sciences University of Antwerp Antwerp Belgium; 2 IMEC-MICT Research Group Department of Communication Sciences, Faculty of Political and Social Sciences Ghent University Ghent Belgium

**Keywords:** COVID-19, SARS-CoV-2, coronavirus, contact tracing, proximity tracing, mHealth, mobile app, user acceptability, surveillance, privacy

## Abstract

**Background:**

In several countries, contact tracing apps (CTAs) have been introduced to warn users if they have had high-risk contacts that could expose them to SARS-CoV-2 and could, therefore, develop COVID-19 or further transmit the virus. For CTAs to be effective, a sufficient critical mass of users is needed. Until now, adoption of these apps in several countries has been limited, resulting in questions on which factors prevent app uptake or stimulate discontinuation of app use.

**Objective:**

The aim of this study was to investigate individuals’ reasons for not using, or stopping use of, a CTA, in particular, the Coronalert app. Users’ and nonusers’ attitudes toward the app’s potential impact was assessed in Belgium. To further stimulate interest and potential use of a CTA, the study also investigated the population’s interest in new functionalities.

**Methods:**

An online survey was administered in Belgium to a sample of 1850 respondents aged 18 to 64 years. Data were collected between October 30 and November 2, 2020. Sociodemographic differences were assessed between users and nonusers. We analyzed both groups’ attitudes toward the potential impact of CTAs and their acceptance of new app functionalities.

**Results:**

Our data showed that 64.9% (1201/1850) of our respondents were nonusers of the CTA under study; this included individuals who did not install the app, those who downloaded but did not activate the app, and those who uninstalled the app. While we did not find any sociodemographic differences between users and nonusers, attitudes toward the app and its functionalities seemed to differ. The main reasons for not downloading and using the app were a perceived lack of advantages (308/991, 31.1%), worries about privacy (290/991, 29.3%), and, to a lesser extent, not having a smartphone (183/991, 18.5%). Users of the CTA agreed more with the potential of such apps to mitigate the consequences of the pandemic. Overall, nonusers found the possibility of extending the CTA with future functionalities to be less acceptable than users. However, among users, acceptability also tended to differ. Among users, functionalities relating to access and control, such as digital certificates or “green cards” for events, were less accepted (358/649, 55.2%) than functionalities focusing on informing citizens about the spread of the virus (453/649, 69.8%) or making an appointment to get tested (525/649, 80.9%).

**Conclusions:**

Our results show that app users were more convinced of the CTA’s utility and more inclined to accept new app features than nonusers. Moreover, nonusers had more CTA-related privacy concerns. Therefore, to further stimulate app adoption and use, its potential advantages and privacy-preserving mechanisms need to be stressed. Building further knowledge on the forms of resistance among nonusers is important for responding to these barriers through the app’s further development and communication campaigns.

## Introduction

Since the emergence of SARS-CoV-2, the subsequent pandemic has been managed by governments worldwide by implementing wide-ranging policies. These include measures that disrupt human mobility, such as full and partial lockdowns, limiting the number of individuals’ physical contacts, and accompanying testing, tracing, and quarantine strategies. Traditionally, contact tracing has been implemented primarily through call centers, where agents interview individuals who have been diagnosed with COVID-19 and people who crossed paths with them [1]. However, contact tracing conducted by a call center has several limitations [2,3]. Therefore, a growing number of countries have developed contact tracing apps (CTAs) that offer users the possibility to keep track of their proximity with other app users and receive warnings if users were close to someone who tested positive for COVID-19 [4]. In most countries where CTAs have been implemented, the use of these apps is voluntary. However, limited uptake levels have been reported [5]. In Europe, uptake levels have been reported as ranging from less than 1% to almost half of the population [6]. Yet, the effectiveness of a CTA depends on the population’s uptake. Modeling studies have quantified the impact of CTA adoption on the spread of the virus. One study found that at least 56% of the population should use a CTA in order to contribute to the mitigation of the pandemic [7]. Even if this threshold is not met, lower uptake levels are able to reduce infection rates and, therefore, use of a CTA could be an effective complement to manual contact tracing. For instance, in a model including 15% of the population using a CTA, exposure notification would reduce the number of infections by 8% [8]. However, the impact of CTAs further depends on measures that are in place, such as nonpharmaceutical measures to mitigate the epidemic (eg, displacement restrictions), the adoption of individual preventive behaviors (eg, physical distancing and isolation compliance for infected individuals), the testing capacity, and easy access to testing facilities to increase early case detection [9].

In Belgium, the CTA Coronalert was launched in September 2020. The app was developed based on the DP-3T (Distributed Privacy Preserving Proximity Tracing) architecture. This was combined with the Exposure Notification interface provided by Google and Apple [10]. The app has been downloaded 2.7 million times, representing almost one-third of Belgian smartphone users [11] (details on how the specific CTA works is summarized in Multimedia Appendix 1). The system offers important privacy safeguards: it only serves to detect close contacts of COVID-19–infected persons, does not track location, and does not link information with personal data [12]. As this system is based on the DP-3T protocol and has also been implemented in a large number of European Union (EU) countries and US states [5], cross-border interoperability has been developed so the app can be used in other countries that use the same system. But for such an app to function optimally, its widespread adoption by the population is crucial.

Previous research focusing on COVID-19 CTAs has concentrated on predictors of app adoption and sociodemographic differences between adopters and nonadopters. Some studies found higher CTA adoption or adoption intention among males, younger respondents, individuals with a higher income, and individuals living in urban areas [13-15]. Studies found that several factors stimulate app uptake, such as current and potential users’ attitudes toward the contribution of the app in diminishing the spread of the virus (ie, perceived usefulness or performance of the app) and positive social influence to use the app (ie, subjective norm). CTAs’ perceived safety and privacy also impacted its use or use intention. Moreover, individuals’ engagement in pandemic-related behavioral adjustments, their trust in government, and their trust in health authorities influenced app uptake [1,15-23]. Respondents who had a personal experience with COVID-19, either as a patient or with relatives who were diagnosed with COVID-19, or those who perceived health consequences in case of infection were more inclined to install the app [15,23,24]. Moreover, research has pointed toward concerns regarding the implementation of CTAs. Users’ perceived security and privacy risks were found to decline app uptake intention [1,14]. Although research has focused on uptake motives and predictors, as well as perceived risks of a CTA, few studies have focused on concerns that fuel nonadoption or discontinuation of use [18,24,25].

Therefore, this paper aims to address an important gap in the literature regarding the nonuse of health-related apps, the relevance of which has become especially apparent in the COVID-19 crisis. As such, this study focuses on potential sociodemographic differences between adopters and nonadopters and reasons for nonuse. More particularly, we focus on the reasons for (1) not downloading the app, (2) having downloaded but not activated the app, and (3) discontinuation of use by uninstalling the app. Moreover, attitudinal differences between nonusers and users were assessed in terms of individual and societal expected outcomes of a CTA.

A second gap that is addressed concerns insight into citizens’ attitudes toward plausible expanding functionalities of CTAs over the course of the pandemic. Therefore, respondents were confronted with potential new features that are not currently integrated in Coronalert but have been implemented in other countries’ CTAs or are being discussed as potential useful additional options to stimulate app uptake and continued use. In this regard, several authors have raised concerns about governments extending personal data collection and use beyond what was originally envisioned in the context of the pandemic (ie, “function creep”) [26,27]. Whereas there are crucial legal aspects connected to the implementation of CTAs and their functionalities [28], the perspective of the end user and the important role of public acceptance cannot be ignored. Therefore, assessing users’ attitudes toward additional data-gathering features of CTAs seems crucial.

Given that population-based research regarding these app functionalities is scarce [29,30], we investigated how users and nonusers differ in their attitudes toward these potential features. For instance, the app could indicate that its holder did not have close contact with another user who tested positive for COVID-19, in order to gain access to public places or other locations. Also, other credentials could be integrated, such as vaccination certificates or results of COVID-19 antibody testing. The verifiers (eg, employers and event organizers) could then ask the holders to present this proof to gain access [31]. Still, the implementation of such “green certificates” have been subject to many criticisms, and several scholars have pointed to the necessary ethical and privacy-related considerations in this regard [31-33].

Therefore, to contribute to the research on digital contact tracing, this study has three main objectives. First, we investigate the thresholds for adoption of CTAs. Second, the potential difference between users and nonusers in terms of CTAs’ perceived impact is examined. Third, we study users’ and nonusers’ openness to potential functionalities that could be included in CTAs.

## Methods

### Procedure and Sample

This study was conducted in Flanders, the Dutch-speaking part of Belgium. An online survey was administered to 18- to 64-year-old respondents. Data were collected between October 30 and November 2, 2020. In that period, the following COVID-19 measures were in place: citizens were allowed to have close contact with a maximum of one person who is not part of one’s own household; citizens were allowed to have private meetings with a maximum of four persons, the same persons within a period of 2 weeks; markets and shops were open; cafés and restaurants were closed, but takeaway and delivery were allowed; telework became the norm for all professional activities that allow it; professional sport competitions could not welcome spectators; indoor events (cultural, religious, etc) could accept a maximum of 200 participants and there were adapted rules for indoor sport activities; and a curfew was in force from 12 AM until 5 AM.

The recruitment of respondents was organized by a professional research agency that manages a panel consisting of 300,000 members in Belgium. Panel members who choose to participate in a survey are not remunerated for their participation but enter in a contest organized by the agency to win vouchers of €50 maximum. Respondents were recruited specifically for the purpose of this study.

A sample of 1850 respondents was recruited with the following eligibility criteria: (1) being a resident of Belgium, (2) being aged between 18 and 64 years, and (3) speaking Dutch. To achieve a heterogeneous sample, we followed a stratified sampling procedure. Based on Belgian federal statistics, we stratified the data a priori regarding gender, age, and education level so that the proportion of the sample’s strata would reflect the proportion of the Flemish population. In total, 8000 panel members were emailed an invitation to participate; the invitation included a short description of the study and a link redirecting respondents to an online survey set up specifically for this study. When 1850 respondents were reached in accordance with the strata, based on gender, age, and education level, data collection was truncated. This was made possible because every panel member’s sociodemographic profile is known by the agency. The researchers had no access to the identity of the participants, and the questionnaire did not request any form of identification that could have inconvenienced respondents or jeopardized their anonymity toward the researchers. Afterward, we confirmed eligibility of the respondents and correspondence with the predefined strata based on sociodemographic variables included in the questionnaire.

After informing the respondents of the study’s objectives and requesting their informed consent, the respondents were confronted with a paragraph briefly explaining the key features of the Coronalert app (ie, the use of Bluetooth to detect proximity and the anonymous disclosure of users’ COVID-19–positive status to other users who have been in their proximity). This study was part of a larger research project concerning predictors of app adoption and use. Prior to the online data collection among the panel members, the survey’s introduction and the whole questionnaire were assessed by three researchers to check the clarity of the questions and the brief explanation.

This study was approved by the Ethical Commission of Ghent University, which supervises the privacy and confidentiality measures taken in each conducted study as well as how data are stored after data collection.

### Measures

Besides the sociodemographic characteristics of gender, age, education level, and employment status, we also questioned the medical condition of respondents. The latter was assessed by asking respondents if they suffered from one or more diseases (eg, heart, lung, or kidney diseases; diabetes; cancer; reduced immune system; and high blood pressure) that could be a risk factor when positive for COVID-19.

The employment categories of Statbel, the Belgian federal statistics institute, were used, based on the International Standard Classification of Occupations. This classification was shortened by grouping several categories, and “flexi-job” was added as a supplementary category, as it is a relatively new employment category.

Respondents were asked about the reasons why they did not install, installed but did not activate, or uninstalled the app. Next, we assessed respondents’ attitudes toward CTAs’ potential impact (8 items). Several statements were presented that were related to the societal and individual implications of mobile contact tracing. Respondents were asked whether they agreed or not with the implications of CTAs, using 5-point Likert scales. In addition, acceptance of potential features and applications of the Coronalert app was measured (11 items using 5-point Likert scales). The submitted options were based on functionalities that are already integrated into specific apps or discussed as potential options [34]; these can be divided in two categories: (1) information and advice and (2) control and access. The first category groups the following advice to users: recognizing symptoms of COVID-19 infection; being informed about infection levels in one’s neighborhood, but also being able to get advice from a health professional; and being able to make an appointment to be tested. The second set of options includes the use of the app as a kind of “corona pass,” to show that one has not been in contact with a person infected with COVID-19 or to allow authorities using the app to check movements of infected persons. Users’ and nonusers’ attitudes were measured on a 5-point Likert scale, ranging from 1 (not agree) to 5 (agree). The study’s questionnaire is included in Multimedia Appendix 2.

### Analytical Strategy

Several analyses were performed to describe differences between users and nonusers of Coronalert, regarding both sociodemographic variables and different attitudes. Prior to the main analyses, all three categories of nonusers (ie, respondents who did not install the app, those who downloaded but did not activate the app, and those who uninstalled the app) were merged into a single group of nonusers. As such, a dichotomous variable of use versus nonuse was created for subsequent analyses. All analyses were performed using SPSS Statistics for Macintosh (version 28; IBM Corp).

First, chi-square analyses and *t* tests were performed to test between-group differences among users and nonusers regarding sociodemographic variables. Afterward, descriptive analyses were performed to assess the different reasons for not using the app. Subsequently, potential differences between users and nonusers were assessed concerning the Coronalert app’s potential impact; users’ and nonusers’ acceptance of new app functionalities was also assessed. Chi-square tests and *t* tests were used for testing categorical and continuous between-group differences, respectively. Cohen *d* was reported and interpreted, along with *P* values, to assess the effect size and presence of significant effects, respectively. Cohen [35] recommends values of 0.10, 0.30, and 0.50 to delimit small, medium, and large effects, respectively.

## Results

### Overview

The study sample’s composition and descriptive statistics are presented in Table 1. In total, 1850 respondents participated in the survey, including 50.4% (933/1850) women. The mean age of the respondents was 45.29 (SD 14.42) years, 39.6% (n=732) had a university or higher education college degree, 39.2% (n=726) had a higher secondary education degree, and 21.2% (n=392) had a lower secondary education degree.

Chi-square tests revealed no significant differences between users and nonusers regarding gender, education level, employment type, and reported health risks. In addition, an independent-samples *t* test indicated no significant differences in terms of age between users and nonusers.

**Table 1:** Study sample and characteristics of users and nonusers of the COVID-19 contact tracing app Coronalert in Belgium.

Characteristic		Total sample (N=1850)	Users of Coronalert (n=649)^a^	Nonusers of Coronalert (n=1201)^a^	Chi-square (*df*)			*t* test (*df*)	*P* value	
Participants, n (%)		1850 (100)	649 (35.1)	1201 (64.9)	N/A^b^			N/A	N/A	
**Gender, n (%)**										
	Male	917 (49.6)	317 (34.6)	600 (65.4)	0.2 (1)	N/A				.65^c^
	Female	933 (50.4)	322 (35.6)	601 (64.4)						
Age in years, mean (SD)		45.29 (14.42)	45.24 (14.68)	45.32 (14.28)	N/A		0.115 (1848)		.47	
**Education level, n (%)**										
	Lower secondary education	392 (21.2)	135 (34.4)	257 (65.5)	0.3 (2)	N/A				.87
	Higher secondary education	726 (39.2)	252 (34.7)	474 (65.3)						
	Higher education	732 (39.6)	262 (35.8)	470 (64.2)						
**Type of employment, n (%)**										
	Worker	439 (23.7)	154 (35.1)	285 (64.9)	0.9 (3)	N/A				.84
	White-collar worker, civil servant, or executive	1120 (60.5)	396 (35.4)	724 (64.6)						
	Self-employed or liberal profession	248 (13.4)	82 (33.1)	166 (66.9)						
	Flexi-job^d^	43 (2.3)	17 (39.5)	26 (60.5)						
**Health risks^e^, n (%)**										
	Yes	694 (37.5)	259 (37.3)	435 (62.7)	3.8 (2)	N/A				.15
	No	1006 (54.4)	333 (33.1)	673 (66.9)						
	I don’t know	150 (8.1)	57 (38.0)	93 (62.0)						

^a^Percentages are based on the total values in the “Total sample” column.

^b^N/A: not applicable; this statistic was not calculated for this item; the *t* test was used for the age variable and the chi-square test was used for all other variables.

^c^Statistics for a set of variables are reported on the top line of that group.

^d^Flexi-job is a specific employment status where people can work additional hours (in the hospitality industry) on favorable terms, even when already retired or employed elsewhere.

^e^Participants with health risks suffer from one or more diseases that can be a risk factor when positive for COVID-19.

### Reasons for Nonuse of Coronalert

In total, 64.9% (1201/1850) of respondents were not using the CTA at the time of the study. The data revealed three types of nonusers: 82.5% (991/1201) had not installed the app, 12.0% (144/1201) downloaded the app but never activated it, and 5.3% (64/1201) had installed the app, but already deleted it from their smartphone. Respondents were questioned about the reasons why they did not install, installed but did not activate, or uninstalled the app. These reasons are summarized in Table 2.

The most important reason for not installing the app was the lack of advantages respondents found in using Coronalert (308/991, 31.1% of the respondents who did not install the app). This was followed by worries about privacy (290/991, 29.3%) and dreading stress when using the app, as reasons for not installing it. Not having a smartphone (183/991, 18.5%) or having an older smartphone model (93/991, 9.4%) were also reasons given by the respondents for not installing the app. A total of 1 in 7 respondents (138/991, 13.9%) saw little value in the app, as they were convinced that they had a low risk of contracting the virus. Reasons for not installing the app that were related to governments’ involvement in the app included worries about how the government would use the collected data (189/991, 19.1%) and that the government would be able to follow users’ movements (80/991, 8.1%). Technical issues, such as experiencing problems when installing the app (46/991, 4.6%), being afraid they would experience difficulties when installing it (63/991, 6.4%), or being afraid that the app would drain the battery (96/991, 9.7%), were less frequently selected as reasons.

A total of 1 in 10 nonusers (144/1201, 11.9%) downloaded the app but did not activate it. The top reasons for these nonactivators included worries about how the government would treat their data (50/144, 34.7%), general privacy concerns (34/144, 23.6%), difficulties in using the app (27/144, 18.8%), or seeing few advantages in using it (16/144, 11.1%).

Another category of respondents deleted the app, although they first decided to install it on their smartphones (64/1201, 5.3% of our nonusers sample). The three most-cited reasons included the following: seeing too few advantages in using it (24/64, 37.5%), experiencing difficulties in using it (16/64, 25.0%), and being afraid the app would impact their smartphone’s battery consumption (12/64, 18.8%).

While a majority of respondents did not install Coronalert, almost 1 in 5 stated that they may decide to install the app in the future (183/991, 18.5%). The main reasons they gave for not yet having adopted this contact tracing technology were related to their smartphone, which was an older model that was not compatible with the app (43/183, 23.5%); not being in the possession of a smartphone (34/183, 18.6%); and having experienced technical issues or not seeing advantages in mobile contact tracing in the context of current COVID-19–related movement restrictions (both 29/183, 15.8%).

**Table 2:** Reasons for nonuse of the COVID-19 contact tracing app Coronalert in Belgium.

Reasons for nonuse of the app	Not installed (n=991), n (%)	Installed, but not activated (n=144), n (%)	Uninstalled (n=64), n (%)
I don’t have a smartphone	183 (18.5)	N/A^a^	N/A
I have an older smartphone	93 (9.4)	N/A	N/A
I experienced a technical problem	46 (4.6)	17 (11.8)	6 (9.4)
I run little risk of contracting the coronavirus	138 (13.9)	12 (8.3)	6 (9.4)
I am afraid that my smartphone battery will drain fast	96 (9.7)	17 (11.8)	12 (18.8)^b^
For me, the app is too difficult to install	63 (6.4)	27 (18.8)^c^	16 (25.0)^c^
I find too few advantages in using the app	308 (31.1)	16 (11.1)	24 (37.5)
I am worried about how the government will use the obtained data	189 (19.1)	50 (34.7)	11 (17.2)
I am afraid that my privacy is not guaranteed when I use the app	290 (29.3)	34 (23.6)	5 (7.8)
I worry that the government will be able to follow my movements	80 (8.1)	11 (7.6)	0 (0)
I do not trust the app	176 (17.8)	6 (4.2)	6 (9.4)
Using the app would cause me stress	208 (21.0)	17 (11.8)	10 (15.6)^d^
I see only few advantages in using the app due to the current measures that make fewer activities outside of home possible	93 (9.4)	27 (18.8)	6 (9.4)

^a^N/A: not applicable; these questions were not submitted to respondents without a smartphone or those with an older smartphone.

^b^The item was adapted to fit the context of stopping the use of Coronalert: “I have the impression that my battery drains more rapidly.”

^c^The item was rephrased as “For me, the app is too difficult to use.”

^d^The item was rephrased as “Using the app stresses me.”

### Differences Between Nonusers and Users as to Coronalert’s Potential Impact

As shown in Table 3, the most important contributions of the app for users were as follows: helping the government in its fight against the pandemic (530/649, 81.7%), a CTA is more rapid than traditional contact tracing in detecting and warning infected users (481/649, 74.1%), the app diminishes the spread of the virus (445/649, 68.6%), the app rapidly alerts users of risky contacts (408/649, 62.9%), and a CTA detects risky contacts while preserving users’ privacy (384/649, 59.2%). Overall, these top five reasons regarding Coronalert’s usefulness were cited less frequently by nonusers of the app, who seemed to be less convinced by the potential impact of the app. An independent-samples *t* test did report a significant difference, with a large effect size (t_1848_=–15.37, *P*<.001 [2-tailed]; Cohen *d*=0.76, 95% CI 0.66-0.86) between app users and nonusers concerning the impact of Coronalert on diminishing the spread of the virus. Users of the app were more convinced of the impact of CTAs than nonusers. Moreover, Coronalert users were more assured than nonusers that the app would inform them more rapidly of potential infections than would traditional contact tracing. This significant difference had a large effect size (t_1624_=–16.99, *P*<.001 [2-tailed]; Cohen *d*=0.78, 95% CI 0.67-0.87). In general, users were more persuaded that a CTA would inform them rapidly if they had a risky contact (t_1848_=–2.55, *P*<.01 [2-tailed]; Cohen *d*=0.13, 95% CI 0.03-0.22). Users were also more convinced that by using a CTA, one would take more precautionary measures not to spread the virus than nonusers, but the difference had a medium effect size (t_1848_=–6.40, *P*<.001 [2-tailed]; Cohen *d*=0.31, 95% CI 0.21-0.41). Users were more strongly convinced that using the app helps the government to fight the virus. The difference between nonusers and users had a strong effect size (t_1716_=–20.81, *P*<.001 [2-tailed]; Cohen *d*=0.92, 95% CI 0.82-1.02). Finally, users were more convinced than nonusers that the CTA respects users’ privacy. However, a small effect size was found (t_1848_=–3.62, *P*<.001 [2-tailed]; Cohen *d*=0.17, 95% CI 0.08-0.27).

**Table 3:** Attitudes toward the potential impact of the COVID-19 contact tracing app Coronalert in Belgium.

Questions and responses				Total sample (N=1850)		Nonusers of Coronalert (n=1201)		Users of Coronalert (n=649)		*t* test (*df*)		*P* value		Cohen *d*
**By using Coronalert, one collaborates in diminishing the spread of the coronavirus**														
	Response score, mean (SD)^a^		N/A^b^		3.03 (1.14)		3.86 (1.01)		–15.37 (1848)^c^		<.001^c^		0.76^c^	
	**Response, n (%)**													
		Not agree	175 (9.5)		159 (13.2)		16 (2.5)							
		Rather disagree	211 (11.4)		161 (13.4)		50 (7.7)							
		Not agree/not disagree	626 (33.8)		488 (40.6)		138 (21.3)							
		Rather agree	520 (28.1)		267 (22.2)		253 (39.0)							
		Agree	318 (17.2)		126 (10.5)		192 (29.6)							
**By using Coronalert, one is more wary when having face-to-face contacts**														
	Response score, mean (SD)		N/A		3.02 (1.15)		3.10 (1.14)		–1.520 (1848)		.13		0.07	
	**Response, n (%)**													
		Not agree	247 (13.4)		171 (14.2)		76 (11.7)							
		Rather disagree	253 (13.7)		156 (13.0)		97 (14.9)							
		Not agree/not disagree	694 (37.5)		466 (38.8)		228 (35.1)							
		Rather agree	476 (25.7)		296 (24.6)		180 (27.7)							
		Agree	180 (9.7)		112 (9.3)		68 (10.5)							
**By using Coronalert, users know rapidly when they have been in contact with someone who is infected with the coronavirus**														
	Response score, mean (SD)		N/A		3.47 (1.12)		3.61 (1.08)		–2.551 (1848)		.01		0.13	
	**Response, n (%)**													
		Not agree	157 (8.5)		116 (9.7)		41 (6.3)							
		Rather disagree	135 (7.3)		79 (6.6)		56 (8.6)							
		Not agree/not disagree	449 (24.3)		305 (25.4)		144 (22.2)							
		Rather agree	811 (43.8)		527 (43.9)		284 (43.8)							
		Agree	298 (16.1)		174 (14.5)		124 (19.1)							
**By using Coronalert, one will take more precautionary measures not to spread the coronavirus**														
	Response score, mean (SD)		N/A		2.82 (1.22)		3.19 (1.15)		–6.396 (1848)		<.001		0.31	
	**Response, n (%)**													
		Not agree	288 (15.6)		233 (19.4)		55 (8.5)							
		Rather disagree	346 (18.7)		213 (17.7)		133 (20.5)							
		Not agree/not disagree	581 (31.4)		403 (33.6)		178 (27.4)							
		Rather agree	447 (24.2)		246 (20.5)		201 (31.0)							
		Agree	188 (10.2)		106 (8.8)		82 (12.6)							
**By using Coronalert, one helps the government in its fight against the coronavirus**														
	Response score, mean (SD)		N/A		3.12 (1.16)		4.09 (0.83)		–20.810 (1716)		<.001		0.92	
	**Response, n (%)**													
		Not agree	150 (8.1)		150 (12.5)		0 (0)							
		Rather disagree	181 (9.8)		143 (11.9)		38 (5.9)							
		Not agree/not disagree	560 (30.3)		479 (39.9)		81 (12.5)							
		Rather agree	592 (32.0)		275 (22.9)		317 (48.8)							
		Agree	367 (19.8)		154 (12.8)		213 (32.8)							


**Coronalert detects contacts with persons who are infected with the coronavirus, respecting the privacy of the app users**														
	Response score, mean (SD)		N/A		3.51 (1.17)		3.71 (1.15)		–3.618 (1848)		<.001		0.17	
	**Response, n (%)**													
		Not agree	166 (9.0)		117 (9.7)		49 (7.6)							
		Rather disagree	64 (3.5)		46 (3.8)		18 (2.8)							
		Not agree/not disagree	615 (33.2)		417 (34.7)		198 (30.5)							
		Rather agree	545 (29.5)		354 (29.5)		191 (29.4)							
		Agree	460 (24.9)		267 (22.2)		193 (29.7)							
**Coronalert is quicker than contact tracing by phone, to check the contacts of people who are infected with the coronavirus**														
	Response score, mean (SD)		N/A		3.28 (1.09)		4.06 (0.85)		–16.985 (1624)		<.001		0.78	
	**Response, n (%)**													
		Not agree	115 (6.2)		115 (9.6)		0 (0)							
		Rather disagree	103 (5.6)		80 (6.7)		23 (3.5)							
		Not agree/not disagree	666 (36.0)		521 (43.4)		145 (22.3)							
		Rather agree	573 (31.0)		321 (26.7)		252 (38.8)							
		Agree	393 (21.2)		164 (13.7)		229 (35.3)							
**Using Coronalert helps to prevent loved ones from being infected with the coronavirus**														
	Response score, mean (SD)		N/A		3.33 (1.23)		3.41 (1.22)		–1.307 (1848)		.19		0.07	
	**Response, n (%)**													
		Not agree	214 (11.6)		146 (12.2)		68 (10.5)							
		Rather disagree	173 (9.4)		108 (9.0)		65 (10.0)							
		Not agree/not disagree	558 (30.2)		377 (31.4)		181 (27.9)							
		Rather agree	539 (29.1)		338 (28.1)		201 (31.0)							
		Agree	366 (19.8)		232 (19.3)		134 (20.6)							

^a^Mean scores were calculated for nonusers and users of the app separately.

^b^N/A: not applicable; mean scores were not calculated for the entire sample.

^c^This value was calculated using the mean scores for users and nonusers of the app and not the frequencies of individual responses.

### Differences Between Nonusers and Users as to Coronalert’s Potential Applications

As highlighted before, almost one-third of respondents (308/991, 31.1%) who did not install the app saw few advantages in using it. Therefore, complementary functionalities that respond to potential users’ needs could stimulate adoption and continued use.

In general, users of Coronalert were more in favor of the potential options that were proposed than respondents who did not use the app (Table 4). Users were most in favor of being informed that they visited a place where one or several persons had later been diagnosed with COVID-19 (547/649, 84.3%), being able to make an appointment to get tested (525/649, 80.9%), getting advice on how to protect oneself (458/649, 70.6%), having contact with a health professional (473/649, 72.9%), receiving statistics about the impact of the virus (eg, number of infections and hospitalizations; 453/649, 69.8%), being informed about the number of infections in one’s neighborhood (438/649, 67.5%), or getting access to a questionnaire to assess COVID-19 symptoms (431/649, 66.4%).

All differences between users and nonusers in their support for the proposed new functionalities were significant, with medium to strong effect sizes. In particular, Coronalert users were significantly more in favor of being informed that they visited a place where one or several persons had later been diagnosed with COVID-19 (t_1565_=–13.62, *P*<.001 [2-tailed]; Cohen *d*=0.54, 95% CI 0.45-0.64), being able to make an appointment with a health professional to get tested (t_1579_=–13.33, *P*<.001 [2-tailed]; Cohen *d*=0.64, 95% CI 0.53-0.73), getting advice on how to protect oneself (t_1418_=–10.03, *P*<.001 [2-tailed]; Cohen *d*=0.37, 95% CI 0.25-0.44), and being able to get in contact with a health professional (t_1438_=–11.43, *P*<.001 [2-tailed]; Cohen *d*=0.54, 95% CI 0.44-0.64). Also, a majority were in favor of viewing statistics about the evolution of the impact of the virus (eg, infections and hospitalizations; t_1527_=–14.91, *P*<.001 [2-tailed]; Cohen *d*=0.69, 95% CI 0.60-0.80), gaining information about the number of infections in one’s neighborhood (t_1239_=–7.55, *P*<.001 [2-tailed]; Cohen *d*=0.38, 95% CI 0.29-0.48), or getting access to a questionnaire to assess COVID-19 symptoms (t_1848_=–9.61, *P*<.001 [2-tailed]; Cohen *d*=0.46, 95% CI 0.37-0.56).

Concerning potential functionalities of Coronalert with a focus on control and access, findings are more mixed. Among users of the app, the implementation of these functionalities seems more debated, as often only half of this group agreed on the future implementation of these functionalities. For example, about half of the users agreed on a “green screen” functionality to access events (358/649, 55.2%), schools (357/649, 55.0%), and offices (322/649, 49.6%). A narrow majority were in favor of using the app to control the whereabouts of people who are infected with COVID-19 (339/649, 52.2%). While overall acceptability of these control functionalities were lower compared to the information-related options, users were still significantly more likely to accept these functionalities compared to nonusers (access to events: t_1248_=–10.73, *P*<.001 [2-tailed]; Cohen *d*=0.53, 95% CI 0.44-0.63; access to schools: t_1282_=–11.06, *P*<.001 [2-tailed]; Cohen *d*=0.55, 95% CI 0.45-0.64; access to offices: t_1225_=–10.55, *P*<.001 [2-tailed]; Cohen *d*=0.53, 95% CI 0.43-0.62; control of whereabouts: t_1179_=–9.20, *P*<.001 [2-tailed]; Cohen *d*=0.47, 95% CI 0.37-0.56).

**Table 4:** Attitudes toward potential applications of the COVID-19 contact tracing app Coronalert in Belgium.

Questions and responses					Total sample (N=1850)		Nonusers of Coronalert (n=1201)		Users of Coronalert (n=649)		*t* test (*df*)		*P* value		Cohen *d*
**Information and advice**															
	**Through a questionnaire that is integrated in the app that questions users about symptoms, you should be able to assess if you are infected with the coronavirus**														
		Response score, mean (SD)^a^		N/A^b^		3.27 (1.12)		3.79 (1.12)		–9.61 (1848)^c^		<.001^c^		0.46^c^	
		**Response, n (%)**													
			Not agree	150 (8.1)		123 (10.2)		27 (4.2)							
			Rather disagree	159 (8.6)		93 (7.7)		66 (10.2)							
			Not agree/not disagree	620 (33.5)		495 (41.2)		125 (19.3)							
			Rather agree	544 (29.4)		317 (26.4)		227 (35.0)							
			Agree	377 (20.4)		173 (14.4)		204 (31.4)							
	**Through the app, you should be able to be informed about how many individuals in your neighborhood are infected with the coronavirus**														
		Response score, mean (SD)		N/A		3.25 (1.14)		3.70 (1.24)		–7.551 (1239)		<.001		0.38	
		**Response, n (%)**													
			Not agree	186 (10.1)		122 (10.2)		64 (9.9)							
			Rather disagree	177 (9.6)		131 (10.9)		46 (7.1)							
			Not agree/not disagree	538 (29.1)		437 (36.4)		101 (15.6)							
			Rather agree	592 (32.0)		342 (28.5)		250 (38.5)							
			Agree	357 (19.3)		169 (14.1)		188 (29.0)							
	**Through the app, you should be able to be informed that you visited a place where one or several persons were present who were infected with the coronavirus**														
		Response score, mean (SD)		N/A		3.55 (1.34)		4.21 (0.93)		–13.62 (1565)		<.001		0.54	
		**Response, n (%)**													
			Not agree	134 (7.2)		116 (9.7)		18 (2.8)							
			Rather disagree	54 (2.9)		37 (3.1)		17 (2.6)							
			Not agree/not disagree	443 (23.4)		366 (30.5)		67 (10.3)							
			Rather agree	693 (37.5)		440 (36.6)		253 (39.0)							
			Agree	536 (29.0)		242 (20.1)		294 (45.3)							
	**Through the app, you should be able to receive advice on how you can better protect yourself against the coronavirus**														
		Response score, mean (SD)		N/A		3.38 (1.8)		3.92 (1.01)		–10.03 (1418)		<.001		0.37	
		**Response, n (%)**													
			Not agree	154 (8.3)		123 (10.2)		31 (4.8)							
			Rather disagree	115 (6.2)		83 (6.9)		32 (4.9)							
			Not agree/not disagree	577 (31.2)		449 (37.4)		128 (19.7)							
			Rather agree	534 (28.9)		311 (25.9)		223 (34.4)							
			Agree	470 (25.4)		235 (19.6)		235 (36.2)							
	**Through the app, you should be able to receive general information on the spread of the coronavirus (eg, weekly averages of infections, hospitalizations, and deaths)**														
		Response score, mean (SD)		N/A		3.16 (1.33)		4.04 (1.13)		–14.91 (1527)		<.001		0.69	
		**Response, n (%)**													
			Not agree	254 (13.7)		226 (18.8)		28 (4.3)							
			Rather disagree	108 (5.8)		76 (6.3)		32 (4.9)							
			Not agree/not disagree	541 (29.2)		405 (33.7)		136 (21.0)							
			Rather agree	411 (22.2)		265 (22.1)		146 (22.5)							
			Agree	536 (29.0)		229 (19.1)		307 (47.3)							
	**Through the app, you should be able to make an appointment to be tested for the coronavirus**														
		Response score, mean (SD)		N/A		3.47 (1.23)		4.20 (1.03)		–13.33 (1579)		<.001		0.64	
		**Response, n (%)**													
			Not agree	176 (9.5)		154 (12.8)		22 (3.4)							
			Rather disagree	90 (4.9)		62 (5.2)		28 (4.3)							
			Not agree/not disagree	416 (22.5)		342 (28.5)		74 (11.4)							
			Rather agree	553 (29.9)		351 (29.2)		202 (31.1)							
			Agree	615 (33.2)		292 (24.3)		323 (49.8)							
	**Through the app, you should be able to get in contact with a health professional to ask advice related to the coronavirus**														
		Response score, mean (SD)		N/A		3.29 (1.30)		3.97 (1.19)		–11.43 (1438)		<.001		0.54	
		**Response, n (%)**													
			Not agree	230 (12.4)		190 (15.8)		40 (6.2)							
			Rather disagree	124 (6.7)		79 (6.6)		45 (6.9)							
			Not agree/not disagree	465 (25.1)		374 (31.1)		91 (14.0)							
			Rather agree	499 (27.0)		310 (25.8)		189 (29.1)							
			Agree	532 (28.8)		248 (20.6)		284 (43.8)							
**Control and access**															
	**Public authorities should be able to follow the whereabouts of people who are infected with the coronavirus**														
		Response score, mean (SD)		N/A		2.63 (1.36)		3.30 (1.56)		–9.20 (1179)		<.001		0.47	
		**Response, n (%)**													
			Not agree	533 (28.8)		388 (32.3)		145 (22.3)							
			Rather disagree	166 (9.0)		98 (8.2)		68 (10.5)							
			Not agree/not disagree	516 (27.9)		419 (34.9)		97 (14.9)							
			Rather agree	281 (15.2)		157 (13.1)		124 (19.1)							
			Agree	354 (19.1)		139 (11.6)		215 (33.1)							
	**The organizer of an event should be able to require participants to show through the Coronalert app on their smartphone that they were not in contact with someone who is infected with the coronavirus**														
		Response score, mean (SD)		N/A		2.74 (1.33)		3.47 (1.43)		–10.73 (1248)		<.001		0.53	
		**Response, n (%)**													
			Not agree	427 (23.1)		332 (27.6)		95 (14.6)							
			Rather disagree	205 (11.1)		124 (10.3)		81 (12.5)							
			Not agree/not disagree	529 (28.6)		414 (34.5)		115 (17.7)							
			Rather agree	334 (18.1)		191 (15.9)		143 (22.0)							
			Agree	355 (19.2)		140 (11.7)		215 (33.1)							
	**An employer should be able to require employees to show through the Coronalert app on their smartphone that they were not in contact with someone who is infected with the coronavirus**														
		Response score, mean (SD)		N/A		2.60 (1.32)		3.32 (1.45)		–10.55 (1225)		<.001		0.53	
		**Response, n (%)**													
			Not agree	499 (27.0)		382 (31.8)		117 (18.0)							
			Rather disagree	193 (10.4)		122 (10.2)		71 (10.9)							
			Not agree/not disagree	553 (29.9)		414 (34.5)		139 (21.4)							
			Rather agree	297 (16.1)		165 (13.7)		132 (20.3)							
			Agree	308 (16.6)		118 (9.8)		190 (29.3)							
	**A school should be able to require students to show through the Coronalert app on their smartphone that they were not in contact with someone who is infected with the coronavirus**														
		Response score, mean (SD)		N/A		2.70 (1.37)		3.46 (1.43)		–11.06 (1282)		<.001		0.55	
		**Response, n (%)**													
			Not agree	461 (24.9)		366 (30.5)		95 (14.6)							
			Rather disagree	197 (10.6)		113 (9.4)		84 (12.9)							
			Not agree/not disagree	510 (27.6)		397 (33.1)		113 (17.4)							
			Rather agree	309 (16.7)		167 (13.9)		142 (21.9)							
			Agree	373 (20.2)		158 (13.2)		215 (33.1)							

^a^Mean scores were calculated for nonusers and users of the app separately.

^b^N/A: not applicable; mean scores were not calculated for the entire sample.

^c^This value was calculated using the mean scores for users and nonusers of the app and not the frequencies of individual responses.

## Discussion

This study found that, one month after its launching, one-third of a stratified sample of the Flemish population used Coronalert. Our analyses showed that there were no significant differences among users and nonusers of the Coronalert app in terms of age, gender, education level, professional activity, and health condition. This contrasts with previous work [18] on the topic and suggests that other, possibly attitudinal, factors are at play. Previous research already highlighted the importance of potential users’ attitudes toward the impact of using a CTA, but also potential concerns about privacy and how users perceive social norms concerning CTA usage [1].

We identified three types of nonusers of the app: those who never installed the app, those who installed but never activated the app, and those who deleted the app after installing. Considering the first group, the most important reasons for not installing the app were a lack of perceived advantages, privacy concerns, and feared stress when using the app. Fewer respondents referred to technical reasons, such as not having a smartphone or having an incompatible or older model, or being convinced that they run little risk of contracting the virus. These results partly correspond, but also contrast, with other research focusing on nonadoption motives. An Australian study found that for those who refused to download the app, privacy concerns constituted the most important reason, followed by technical problems [25]. A multi-country study confirmed that one of the main factors that may hinder app uptake are concerns over privacy and cybersecurity [17]. In research conducted in Switzerland and France, the lack of usefulness was the most important reason, but privacy and security concerns were also mentioned as important reasons [18,36]. Technical reasons were less stressed by this study’s respondents, but were highlighted in other studies [18,25]. Nevertheless, making the app compatible with older smartphones could be important to enhance its use, as 9.4% (93/991) of this study’s respondents had compatibility issues. Still, an important proportion of respondents (183/991, 18.5%) did not possess a smartphone and, therefore, were excluded from using this contact tracing technology. To be able to reach members of this population who are interested in digital contact tracing but do not possess a compatible smartphone, an adapted contact tracing system could be proposed that complements the use of CTAs, namely Bluetooth tokens [37,38]. This system could help cover people without a smartphone or those who prefer not to use a CTA [39].

In contrast with the study by von Wyl et al [18] among Swiss citizens, more Belgian respondents were concerned about the app’s battery use. Moreover, lack of trust in government was expressed by a limited number of Swiss respondents. By contrast, more Belgian respondents feared the government’s use of the collected data (189/991, 19.1%). In addition, almost one-fifth of nonusers (176/991, 17.8%) stated that they do not trust the app. Concerns of government surveillance at the end of the pandemic was also an important reason for not installing the app in a five-country survey [17]. In other words, nonadopters need to be convinced of how users’ privacy is protected. Stressing the data-minimizing solution that has been adopted not only protects users’ privacy rights but also stimulates broader support in the population [40]. Therefore, increasing the readability of the privacy policy could reassure potential users and increase app adoption [41].

Another important reason given by nonusers was feared stress when using the app (208/991, 21.0% of current nonusers). Therefore, clear explanations should be given in the app, as well as in video animations on the app’s website, regarding the steps to take when confronted with a message that one has had a high-risk exposure. At this stressful moment, users need assistance in carefully taking the right steps to get tested and engage in protective measures. However, user statistics of Coronalert revealed that 37% of all app users who received a positive COVID-19 test result—in total, some 20,000 users—confirmed their status through the app, which automatically and anonymously informed close contacts that they have been near someone who has tested positive [42]. In other words, almost two-thirds did not engage in this essential step to warn other users. Therefore, more accompaniment is needed when users are confronted with this stressful news, to encourage them to engage in warning other users. In general, more information is needed about how the app functions, as other research found there are some important misconceptions about the possibilities and limits of contact tracing technology [25].

The study also found out that some potential users still need to be convinced of the app’s potential impact. In total, 31.1% (308/991) of individuals who did not install the app saw limited advantages in using it. Although some contact restricting measures were in place when the survey was fielded, the app could still prove its usefulness in tracing risky contacts in shops and other public places that were open. Stressing the potential impact of the app is important to augment individuals’ uptake intention. Previous research found that the strongest predictor of app use intention among potential users was their expectations concerning the performance of the app to augment their knowledge of potential confrontation with a COVID-19–positive contact and how it could help circumvent the spread of the virus [1]. Therefore, testimonials from users and influencers on general media and social media could be used to inform nonusers about their positive experience with the app [1,18,39,43]. In Belgium, public broadcasters and other media have explained Coronalert’s functioning. However, when launching the app and at the time of this survey, only textual information was included on the website and on the app explaining the app’s functioning. No video animations were available on the website or on the app that clearly explained how Coronalert functions [44]. This contrasts with other countries, where video animations clearly explain how the implemented CTA works and also touch on sensitive issues, such as privacy [45].

This study’s results further show that a small part of the sample (144/1850, 7.8%) have installed the app on their smartphone, but eventually decided not to activate it. This group, who were first convinced to download Coronalert but then hesitated to use it, could be further informed about the advantages of app use. Additionally, some of their concerns could be countered by explaining how the app protects users’ privacy by not identifying nor individually locating users; at the same time, the advantages one has in using the app could be stressed in order to dispel their doubts. Moreover, Coronalert and other CTAs are increasingly interoperable in EU member states [46]. This could be stressed as an important advantage when traveling.

Another category of respondents first downloaded the app but eventually uninstalled it from their smartphone (64/1850, 3.5%). They gave similar reasons to those of the nonadopters. For instance, respondents who uninstalled the app stated that they experienced difficulties using the app. It would, therefore, be important for app developers to gain in-depth insight into the issues that former users have experienced. Moreover, additional usability research could be conducted, as previous research among potential users found issues related to the understandability of CTAs, doubts concerning their usefulness and privacy, and which follow-up actions were expected after a risk exposure notification [47]. Moreover, previous research analyzing media content concerning the implementation of CTAs has identified thresholds and challenges experienced by users and showed the need to intensify communication about the benefits of using the apps [48]. By scraping social media and analyzing app users’ reviews, comments, and reported technical issues, developers could collect input to address reported issues and further develop CTA functionalities [4,49]. Also, by conducting in-depth interviews with potential users and analyzing media coverage on CTAs, the framing of the app’s functionalities and discussed issues can be detected [50].

The study further found that nonusers were significantly less convinced than users of several potential contributions of the app during this pandemic. While a majority of users (445/649, 68.6%) were convinced that it can contribute to diminishing the spread of the virus, only one-third (393/1201, 32.7%) of nonusers agreed. Users were also more convinced that the app helps the government in its fight and is quicker than traditional contact tracing, while, at the same time, respecting individuals’ privacy. This corroborates the already-stressed importance of making the impact of using Coronalert more concrete and visible and, at the same time, showing how the system respects users’ privacy. Communication campaigns could stress specific individual and societal advantages of contact tracing. Moreover, research into the reasons that could trigger nonusers to adopt the app could be used to lower thresholds for nonadopters. For instance, vulnerable groups (eg, senior citizens and individuals with comorbidities) and groups with a high potential to spread the virus, because they are frequently in contact with other people outside their household, could be targeted by specific campaigns to drive them to adopt the app [51].

Finally, this study also assessed the potential support for additional functionalities. Among both users and nonusers, functionalities that focus on information were considered more acceptable than options concerning control and access. For example, users were most in favor of being informed that they had visited a place where people were present who had been diagnosed with COVID-19. This would need adaptations of the current system, as location is not recorded. An alternative would be to have check-ins in public places, so visitors would be informed if they have been in proximity with confirmed COVID-19 cases [52]. Moreover, a majority of users and nonusers were in favor of expanding the app’s mobile health functionalities, by including more information and advice on how to prevent infection and recognize symptoms as well as being able to get in touch with a health professional for advice.

While the possibility of using the app as a green card for events, school, and workplace access was most favored in the “control and access” category, overall acceptance was rather low. Among the users, only half of the respondents agreed that this kind of functionality should be implemented. Among nonusers, acceptance was even lower, with a big majority of the respondents indicating that they are not in favor of this option. These results correspond to a US study that found that a minority of young adults were willing to accept digital surveillance prior to participating in activities in public places (eg, concerts and restaurants) [53]. The EU Digital COVID Certificate includes information on citizens’ vaccination, test, and recovery status [54]. However, our results indicate that public support among Flemish citizens for such implementations is low. In sum, governments and app developers need to strike the right balance between finding appealing new functionalities that stimulate app uptake and sustained use, while addressing privacy and other issues voiced by potential users [3].

Several limitations apply to this study. First, although our sample’s strata were based on the proportions reported by the country’s official statistics concerning age, gender, and education level, we may have missed specific groups, more particularly, individuals who are disadvantaged in terms of income, health status, or other characteristics. Relatedly, it is possible that our sample was prone to self-selection bias, given that members of the panel were free to participate in the study. However, we aimed to counter this bias by relying on a stratified sampling procedure, following federal statistics of the sociodemographic profile of Belgian citizens. Second, as the pandemic and subsequent measures still develop, further research is needed on app use intention, actual usage, and discontinuation of use in time periods where more or less restricting measures are in place. Therefore, it could be important to conduct longitudinal research or comparative research between countries that have different levels of COVID-19–related measures, as motivations to adopt CTAs may fluctuate depending on the measures in place that limit social contact. Further comparative research could also be encouraged to address the reasons for nonadoption or discontinuation of use. By conducting research in countries with different political systems, the role of trust in government and other institutions involved in the development and deployment of CTAs could be further investigated [17]. Finally, this study focused on public support for new functionalities in one country. Future research might investigate which specific combination of functionalities works best in which countries and among which specific target groups [3].

## Appendix

Multimedia Appendix 1 How the contact tracing app (CTA) Coronalert works.

Multimedia Appendix 2 Questionnaire concerning Coronalert.

## Abbreviations

CTA: contact tracing app
DP-3T: Distributed Privacy Preserving Proximity Tracing
EU: European Union

## References

[1] Walrave M, Waeterloos C, Ponnet K (2021). Ready or not for contact tracing? Investigating the adoption intention of COVID-19 contact-tracing technology using an extended unified theory of acceptance and use of technology model. Cyberpsychol Behav Soc Netw.

[2] Keeling MJ, Hollingsworth TD, Read JM (2020). Efficacy of contact tracing for the containment of the 2019 novel coronavirus (COVID-19). J Epidemiol Community Health.

[3] Weiß JP, Esdar M, Hübner U (2021). Analyzing the essential attributes of nationally issued COVID-19 contact tracing apps: Open-source intelligence approach and content analysis. JMIR Mhealth Uhealth.

[4] Elkhodr M, Mubin O, Iftikhar Z, Masood M, Alsinglawi B, Shahid S, Alnajjar F (2021). Technology, privacy, and user opinions of COVID-19 mobile apps for contact tracing: Systematic search and content analysis. J Med Internet Res.

[5] MIT Technology Review Covid Tracing Tracker. Google Docs.

[6] LibertiesEU (2021). COVID-19 contact tracing apps in the EU. Liberties.

[7] Hinch R, Probert W, Nurtay A, Kendall M, Wymant C, Hall M, Lythgoe K, Bulas Cruz A, Zhao L, Stewart A, Ferretti L, Parker M, Meroueh A, Mathias B, Stevenson S, Montero D, Warren J, Mather NK, Finkelstein A, Abeler-Dörner L, Bonsall D, Fraser C (2020). Effective configurations of a digital contact tracing app: A report to NHSX. The Conversation Canada.

[8] Abueg M, Hinch R, Wu N, Liu L, Probert W, Wu A, Eastham P, Shafi Y, Rosencrantz M, Dikovsky M, Cheng Z, Nurtay A, Abeler-Dörner L, Bonsall D, McConnell MV, O'Banion S, Fraser C (2021). Modeling the effect of exposure notification and non-pharmaceutical interventions on COVID-19 transmission in Washington state. NPJ Digit Med.

[9] Moreno López JA, Arregui García B, Bentkowski P, Bioglio L, Pinotti F, Boëlle P, Barrat A, Colizza V, Poletto C (2021). Anatomy of digital contact tracing: Role of age, transmission setting, adoption, and case detection. Sci Adv.

[10] Corona App Task Force (2020). Coronalert: A distributed privacy-friendly contact tracing app for Belgium. Version 1.3. Departement Elektrotechniek, KU Leuven.

[11] Vanmeldert D, Verheyden T, Aerts B, Dillen M, Marselis D, Sehl M, Auffret S (2021). Data Covid-19 contact tracing apps. The Investigative Desk.

[12] Sciensano (2021). How we protect your data and privacy?. Coronalert.

[13] Horstmann KT, Buecker S, Krasko J, Kritzler S, Terwiel S (2020). Short report: Who does or does not use the "Corona-Warn-App" and why?. Eur J Public Health.

[14] Li T, Cobb C, Yang J, Baviskar S, Agarwal Y, Li B, Bauer L, Hong JI (2021). What makes people install a COVID-19 contact-tracing app? Understanding the influence of app design and individual difference on contact-tracing app adoption intention. Pervasive Mob Comput.

[15] Hargittai E, Redmiles EM, Vitak J, Zimmer M (2020). Americans’ willingness to adopt a COVID-19 tracking app. First Monday.

[16] Walrave M, Waeterloos C, Ponnet K (2020). Adoption of a contact tracing app for containing COVID-19: A health belief model approach. JMIR Public Health Surveill.

[17] Altmann S, Milsom L, Zillessen H, Blasone R, Gerdon F, Bach R, Kreuter F, Nosenzo D, Toussaert S, Abeler J (2020). Acceptability of app-based contact tracing for COVID-19: Cross-country survey study. JMIR Mhealth Uhealth.

[18] von Wyl V, Höglinger M, Sieber C, Kaufmann M, Moser A, Serra-Burriel M, Ballouz T, Menges D, Frei A, Puhan MA (2021). Drivers of acceptance of COVID-19 proximity tracing apps in Switzerland: Panel survey analysis. JMIR Public Health Surveill.

[19] Saw YE, Tan EY, Liu JS, Liu JC (2021). Predicting public uptake of digital contact tracing during the COVID-19 pandemic: Results from a nationwide survey in Singapore. J Med Internet Res.

[20] Oldeweme A, Märtins J, Westmattelmann D, Schewe G (2021). The role of transparency, trust, and social influence on uncertainty reduction in times of pandemics: Empirical study on the adoption of COVID-19 tracing apps. J Med Internet Res.

[21] Tomczyk S, Barth S, Schmidt S, Muehlan H (2021). Utilizing health behavior change and technology acceptance models to predict the adoption of COVID-19 contact tracing apps: Cross-sectional survey study. J Med Internet Res.

[22] Ezzaouia I, Bulchand-Gidumal J (2021). A model to predict users' intentions to adopt contact-tracing apps for prevention from COVID-19. Proceedings of the 28th Annual International eTourism Conference (ENTER21).

[23] Guillon M, Kergall P (2020). Attitudes and opinions on quarantine and support for a contact-tracing application in France during the COVID-19 outbreak. Public Health.

[24] Utz C, Becker S, Schnitzler T, Farke F, Herbert F, Schaewitz L, Degeling M, Dürmuth M (2021). Apps against the spread: Privacy implications and user acceptance of COVID-19-related smartphone apps on three continents. Proceedings of the CHI Conference on Human Factors in Computing Systems.

[25] Thomas R, Michaleff ZA, Greenwood H, Abukmail E, Glasziou P (2020). Concerns and misconceptions about the Australian Government's COVIDSafe app: Cross-sectional survey study. JMIR Public Health Surveill.

[26] Vitak J, Zimmer M (2020). More than just privacy: Using contextual integrity to evaluate the long-term risks from COVID-19 surveillance technologies. Soc Media Soc.

[27] Madianou M (2020). A second-order disaster? Digital technologies during the COVID-19 pandemic. Soc Media Soc.

[28] Bradford L, Aboy M, Liddell K (2020). COVID-19 contact tracing apps: A stress test for privacy, the GDPR, and data protection regimes. J Law Biosci.

[29] Nehme M, Stringhini S, Guessous I, SEROCoV-Pop Study Team (2020). Perceptions of immunity and vaccination certificates among the general population: A nested study within a serosurvey of anti-SARS-CoV-2 antibodies (SEROCoV-POP). Swiss Med Wkly.

[30] Kowalewski M, Herbert F, Schnitzler T, Dürmuth M Proof-of-vax: Studying user preferences and perception of Covid vaccination certificates. ArXiv. Preprint posted online on June 22, 2021.

[31] Eisenstadt M, Ramachandran M, Chowdhury N, Third A, Domingue J (2020). COVID-19 antibody test/vaccination certification: There's an app for that. IEEE Open J Eng Med Biol.

[32] Marhold K, Fell J (2021). Electronic vaccination certificates: Avoiding a repeat of the contact-tracing 'format wars'. Nat Med.

[33] Mbunge E, Fashoto SG, Batani J (2021). COVID-19 digital vaccination certificates and digital technologies: Lessons from digital contact tracing apps. SSRN J.

[34] World Health Organization (2020). Digital Tools for COVID-19 Contact Tracing: Annex: Contact Tracing in the Context of COVID-19.

[35] Cohen J (1988). Statistical Power Analysis for the Behavioral Sciences. 2nd edition.

[36] Montagni I, Roussel N, Thiébaut Rodolphe, Tzourio C (2021). Health care students' knowledge of and attitudes, beliefs, and practices toward the French COVID-19 app: Cross-sectional questionnaire study. J Med Internet Res.

[37] (2020). Anonymous COVID-19 contact tracing using physical tokens. EIT Digital.

[38] (2021). TraceTogether Token. TraceTogether.

[39] Seto E, Challa P, Ware P (2021). Adoption of COVID-19 contact tracing apps: A balance between privacy and effectiveness. J Med Internet Res.

[40] Abeler J, Bäcker M, Buermeyer U, Zillessen H (2020). COVID-19 contact tracing and data protection can go together. JMIR Mhealth Uhealth.

[41] Zhang M, Chow A, Smith H (2020). COVID-19 contact-tracing apps: Analysis of the readability of privacy policies. J Med Internet Res.

[42] Lhuillier V (2021). Coronalert, l'application beaucoup téléchargée mais peu utilisée. BX1.

[43] Abeler J (2020). Peer-to-peer marketing of a Covid-19 contact-tracing app. Version 1. Open Science Framework.

[44] Walrave M, Baert E, Ponnet K (2021). How Do We Stand Towards Digital Contact Tracing? Insight Into the Acceptance and Use of the Coronalert App [Article in Dutch].

[45] Protecting your privacy and security. NHS COVID-19 App Support.

[46] Mobile contact tracing apps in EU Member States. European Commission.

[47] Bente BE, van 't Klooster JWJR, Schreijer MA, Berkemeier L, van Gend JE, Slijkhuis PJH, Kelders SM, van Gemert-Pijnen JEWC (2021). The Dutch COVID-19 contact tracing app (the CoronaMelder): Usability study. JMIR Form Res.

[48] von Wyl V (2021). Challenges for nontechnical implementation of digital proximity tracing during the COVID-19 pandemic: Media analysis of the SwissCovid app. JMIR Mhealth Uhealth.

[49] Ahmad K, Alam F, Qadir J, Qolomany B, Khan I, Khan T, Suleman M, Said N, Hassan S, Gul A, Al-Fuqaha A Sentiment analysis of users? Reviews on COVID-19 contact tracing apps with a benchmark dataset. ArXiv. Preprint posted online on March 1, 2021.

[50] Zimmermann BM, Fiske A, Prainsack B, Hangel N, McLennan S, Buyx A (2021). Early perceptions of COVID-19 contact tracing apps in German-speaking countries: Comparative mixed methods study. J Med Internet Res.

[51] Blom AG, Wenz A, Cornesse C, Rettig T, Fikel M, Friedel S, Möhring K, Naumann E, Reifenscheid M, Krieger U (2021). Barriers to the large-scale adoption of a COVID-19 contact tracing app in Germany: Survey study. J Med Internet Res.

[52] Protect your visitors and staff. NHS COVID-19 App Support.

[53] Maytin L, Maytin J, Agarwal P, Krenitsky A, Krenitsky J, Epstein RS (2021). Attitudes and perceptions toward COVID-19 digital surveillance: Survey of young adults in the United States. JMIR Form Res.

[54] (2021). EU Digital COVID Certificate: European Parliament and Council reach agreement on Commission proposal. European Commission.

