# Supplementary material for: Reasons for Nonuse, Discontinuation of Use, and Acceptance of Additional Functionalities of a COVID-19 Contact Tracing App: Cross-sectional Survey Study
Source: JMIR Public Health Surveill. 2022 Jan 14;8(1):e22113. doi: 10.2196/22113 (PMC8763311; doi:10.2196/22113)
Supplement: Multimedia Appendix 1 [file publichealth_v8i1e22113_app1.docx]

How the contact tracing app (CTA) Coronalert works.

The CTA works as follows. The app generates anonymous temporary IDs (every 10 to 20 minutes). Each phone running the app exchanges the anonymous IDs via Bluetooth with other phones using the app who are close to each other (less than 1.5m away for at least 15 minutes). These IDs are stored on a user’s smartphone for fourteen days. When a user tests positive for COVID-19, the app will ask to share the positive test result and anonymous ID with the central database. This request is voluntary. All smartphones on which the Coronalert app is installed connect daily to the database. The devices check the anonymous IDs of the app users who have shared their positive test result and ID with the database, and a possible match is searched for. This control is completely decentralized (on the user’s device). If no match with the IDs is found, a green screen is showed (i.e., low risk). If there is a match, users see a red screen (i.e., high risk). A warning will then appear that the user has had a risk contact, namely a close contact with a Coronalert user who has tested positive for the coronavirus. However, no information is released about who is infected, or where and when this contact took place. When there is a positive match, the user is advised to self-isolate immediately and get tested when showing symptoms. A warning by the app may thus prevent a user to contaminate others, when conforming to the appropriate measures [10].
