# Supplementary material for: Reasons for Nonuse, Discontinuation of Use, and Acceptance of Additional Functionalities of a COVID-19 Contact Tracing App: Cross-sectional Survey Study
Source: JMIR Public Health Surveill. 2022 Jan 14;8(1):e22113. doi: 10.2196/22113 (PMC8763311; doi:10.2196/22113)
Supplement: Multimedia Appendix 2 [file publichealth_v8i1e22113_app2.docx]

Dear Respondent,

Thank you for participating in this survey.

The spread of Covid-19 or the coronavirus, has had a strong impact on our daily lives in recent months. Various measures are being taken by the government. Technology is also being used to reduce the spread of the coronavirus.

In this study, we want to ask your opinion about Coronalert, the app that alerts you if you had close contact with another app user who has contracted the coronavirus.

The results of this study will lead to new insights that are scientifically important and can inspire policy.

Good to know if you want to participate:

- The questionnaire is anonymous, we ask for some personal data such as your year of birth, gender, family situation, but these data do not allow in any way to identify you.
- Your answers will be kept confidential and used for scientific purposes by researchers at the University of … and the University of … .
- You have the right to stop completing this questionnaire at any time, if you wish so.
- After completing it, you have the right to ask for additional information about the processing of your data (mail to …@uantwerpen.be).

I hereby confirm that I have been adequately informed about the study and that I have understood the information. The investigators have provided me with sufficient information regarding the study.

I hereby certify that I

1) have been informed of the purpose of the study;

2) participate in the scientific research of my own free will;

3) am aware that the results will be stored, processed, reported and shared in an anonymous way;

4) am aware of the possibility to discontinue participation in the study at any time;

5) know that more information about the study can be obtained on request.

Do you agree to the collection, processing, use and sharing of your data in an anonymous way in our scientific research?

I agree (1) - I disagree (2)

We start with some questions about you.

What is your gender? Male (1) - Female (2) - Other (3)

What is your year of birth? . . . .

What is the zip code of the town/city where you live? . . . .

What is currently your highest obtained diploma?

No diploma, a diploma of primary education or a diploma of lower secondary education (= third year of secondary education completed) (1)

A diploma of secondary education (= sixth or seventh year of secondary education completed) (2)

Higher education diploma (= higher education completed) (3)

What is your current marital status? Unmarried (1) - Married (2) - Widowed (3) - Divorced (4)

What type of job or jobs do you perform?

Multiple answers possible.

Employee: laborer (1)

Employee: clerk, official or executive (2)

Self-employed or liberal profession (3)

Flexi-job (4)

Other (5)

Do you suffer from one or more conditions that may be a risk factor in coronavirus infection (e.g., heart, lung, kidney disease, diabetes, cancer diagnosis, weakened immune system, high blood pressure, etc.)?

Yes (1) - No (2) - I don't know (3)

Coronalert is a free app launched in Belgium in the fight against the coronavirus.

*What is the purpose of the app?*

When you use the app, you will be alerted if you had close contact with another app user who has contracted the coronavirus.

If you as a user tested positive for the coronavirus, then other app users with whom you came in contact, will be alerted.

These alerts are done completely anonymously. App users do not find out the identity of which person led to the infection, nor where and when this contact took place.

*How does the app work?*

You activate the app on your smartphone and turn on your Bluetooth.

When you are in close contact with another app user, at least 15 minutes within 1.5 meters of each other, both cell phones exchange anonymous codes (=combinations of numbers and letters).

Your cell phone stores all anonymous codes of users with whom you were in close contact. These codes are stored on the cell phone for 14 days.

When an app user is infected, that user shares with a central database the anonymous codes that his/her cell phone has sent in the past days to the cell phones of people with whom he/she was in close contact.

Continuously, the anonymous codes your cell phone received are compared with the codes in a central database. When this comparison shows that you were in contact with someone infected with the coronavirus, you receive a warning message on your cell phone.

This warning notifies you that you may have been in contact with someone infected with the coronavirus. You are not given any information about the identity of this person, nor when and where this contact took place.

*What if you receive a warning message?*

The warning message gives you guidelines:

- isolate yourself (=home quarantine) and
- get yourself tested for the coronavirus.

If the test is positive and you are infected with the coronavirus, you will be asked via the app to share your list of anonymous codes (of app users with whom you had close contact) with the central database. These app users will then receive an anonymous warning, namely that they may have been in contact with a person infected with the coronavirus.

Do you use the app Coronalert?

I use the app (1)

I have not installed the app on my smartphone (2)

I have installed the app but have not opened/started it yet (3)

I have installed the app but have uninstalled it in the meantime (4)

Why have you not installed the app Coronalert? Please indicate one or more reasons.

I don’t have a smartphone

I have an older smartphone

I experienced a technical problem

I run little risk of contracting the coronavirus

I am afraid that my smartphone battery will drain fast

For me, the app is too difficult to install

I find too few advantages in using the app

I am worried about how the government will use the obtained data

I am afraid that my privacy is not guaranteed when I use the app

I worry that the government will be able to follow my movements

I do not trust the app

Using the app would cause me stress

I see only few advantages in using the app due to the current measures that make fewer activities outside of home possible

Why have you not yet opened/activated Coronalert? Please indicate one or more reasons.

I experienced a technical problem

I run little risk of contracting the coronavirus

I am afraid that my smartphone battery will drain fast

For me the app is too difficult to use

I find too few advantages in using the app

I am worried about how the government will use the obtained data

I am afraid that my privacy is not guaranteed when I use the app

I worry that the government will be able to follow my movements

I do not trust the app

Using the app would cause me stress

I see only few advantages in using the app due to the current measures that make fewer activities outside of home possible

Why did you stop using Coronalert by deleting the app from your smartphone? Please indicate one or more reasons.

I experienced a technical problem

I run little risk of contracting the coronavirus

I have the impression that my battery drains more rapidly

For me, the app is too difficult to use

I find too little advantages in using the app

I am worried about how the government will use the obtained data

I am afraid that my privacy is not guaranteed when I use the app

I worry that the government will be able to follow my movements

I do not trust the app

Using the app stresses me

I see only few advantages in using the app due to the current measures that make fewer activities outside of home possible

To what extent do you agree with the following statements concerning use impact?

Not agree

Rather disagree

Not agree/not disagree

Rather agree

Agree

By using Coronalert, one collaborates in diminishing the spread of the coronavirus

By using Coronalert, one is more wary when having face-to-face contacts

By using Coronalert, users know rapidly when they have been in contact with someone who is infected with the coronavirus

By using Coronalert, one will take more precautionary measures not to spread the coronavirus

By using Coronalert, one helps the government in its fight against the coronavirus

Coronalert detects contacts with persons who are infected with the coronavirus, respecting the privacy of the app users

Coronalert is quicker than contact tracing by phone, to check the contacts of people who are infected with the coronavirus

Using Coronalert helps to prevent that loved ones are infected with the coronavirus

To what extent do you agree with the following statements concerning potential applications?

Not agree

Rather disagree

Not agree/not disagree

Rather agree

Agree

Through a questionnaire that is integrated in the app that questions users about symptoms, you should be able to assess if you are infected by the coronavirus.

Through the app, you should be able to be informed about how many individuals in your neighborhood are infected with the coronavirus.

Through the app, you should be able to be informed that you visited a place where one or several persons were present who were infected with the coronavirus.

Through the app, you should be able to receive advice on how you can better protect yourself against the coronavirus.

Through the app, you should be able to receive general information on the spread of the coronavirus (e.g., weekly averages of infections, hospitalizations, deaths).

Through the app, you should be able to make an appointment to be tested for the coronavirus.

Through the app, you should be able to get in contact with a health professional to ask advice related to the coronavirus.

Public authorities should be able to follow the whereabouts of people who are infected with the coronavirus.

The organizer of an event should be able to require participants to show through the Coronalert app on their smartphone that they were not in contact with someone who is infected with the coronavirus.

An employer should be able to require employees to show through the Coronalert app on their smartphone that they were not in contact with someone who is infected with the coronavirus.

A school should be able to require students to show through the Coronalert app on their smartphone that they were not in contact with someone who is infected with the coronavirus.

Thank you very much for your cooperation in this study.

If you have any questions about this study, you can reach the researchers at …@uantwerpen.be
